# Association of caffeine consumption with cerebrospinal fluid biomarkers in mild cognitive impairment and Alzheimer's disease: A BALTAZAR cohort study

**DOI:** 10.1002/alz.14169

**Published:** 2024-08-04

**Authors:** David Blum, Emeline Cailliau, Hélène Béhal, Jean‐Sébastien Vidal, Constance Delaby, Luc Buée, Bernadette Allinquant, Audrey Gabelle, Stéphanie Bombois, Sylvain Lehmann, Susanna Schraen‐Maschke, Olivier Hanon, BALTAZAR study group, Olivier Hanon, Frédéric Blanc, Yasmina Boudali, Audrey Gabelle, Jacques Touchon, Marie‐Laure Seux, Hermine Lenoir, Catherine Bayle, Stéphanie Bombois, Christine Delmaire, Xavier Delbeuck, Florence Moulin, Emmanuelle Duron, Florence Latour, Matthieu Plichart, Sophie Pichierri, Galdric Orvoën, Evelyne Galbrun, Giovanni Castelnovo, Lisette Volpe‐Gillot, Florien Labourée, Pascaline Cassagnaud, Claire Paquet, Françoise Lala, Bruno Vellas, Julien Dumurgier, Anne‐Sophie Rigaud, Christine Perret‐Guillaume, Eliana Alonso, Foucaud du Boisgueheneuc, Laurence Hugonot‐Diener, Adeline Rollin‐Sillaire, Olivier Martinaud, Clémence Boully, Yann Spivac, Agnès Devendeville, Joël Belmin, Philippe Robert, Thierry Dantoine, Laure Caillard, David Wallon, Didier Hannequin, Nathalie Sastre, Sophie Haffen, Anna Kearney‐Schwartz, Jean‐Luc Novella, Vincent Deramecourt, Valérie Chauvire, Gabiel Abitbol, Nathalie Schwald, Caroline Hommet, François Sellal, Marie‐Ange Cariot, Mohamed Abdellaoui, Sarah Benisty, Salim Gherabli, Pierre Anthony, Frédéric Bloch, Nathalie Charasz, Sophie Chauvelier, Jean‐Yves Gaubert, Guillaume Sacco, Olivier Guerin, Jacques Boddaert, Marc Paccalin, Marie‐Anne Mackowiak, Marie‐Thérèse Rabus, Valérie Gissot, Athanase Benetos, Candice Picard, Céline Guillemaud, Gilles Berrut, Claire Gervais, Jacques Hugon, Jean‐Marc Michel, JeanPhilippe David, Marion Paulin, Pierre‐Jean Ousset, Pierre Vandel, Sylvie Pariel, Vincent Camus, Anne Chawakilian, Léna Kermanac'h, Anne‐Cécile Troussiere, Cécile Adam, Diane Dupuy, Elena Paillaud, Hélène Briault, Isabelle Saulnier, Karl Mondon, Marie‐Agnès Picat, Marie Laurent, Olivier Godefroy, Rezki Daheb, Stéphanie Libercier, Djamila Krabchi, Marie Chupin, JeanSébastien Vidal, Edouard Chaussade, Christiane Baret‐Rose, Sylvain Lehmann, Bernadette Allinquant, Susanna Schraen‐Maschke

**Affiliations:** ^1^ University of Lille, Inserm, CHU Lille UMR‐S1172 Lille Neuroscience & Cognition (LilNCog) Lille France; ^2^ Alzheimer and Tauopathies LabEx DISTALZ Lille France; ^3^ Department of Biostatistics CHU Lille Lille France; ^4^ Université Paris Cité INSERM U1144, GHU APHP Centre Hopital Broca, Memory Resource and Research Centre de Paris‐Broca‐Ile de France Paris France; ^5^ Laboratoire et Plateforme de Protéomique Clinique Université de Montpellier INM INSERM, IRMB CHU de Montpellier, 80 av Fliche Montpellier France; ^6^ Sant Pau Memory Unit Hospital de la Santa Creu i Sant Pau ‐ Biomedical Research Institute Sant Pau ‐ Universitat Autònoma de Barcelona Barcelona Spain; ^7^ Université Paris Cité Institute of Psychiatry and Neuroscience, Inserm, UMR‐S 1266 Paris France; ^8^ Université de Montpellier CHU Montpellier Memory Research and Resources Center Department of Neurology, Inserm INM NeuroPEPs Team Excellence Center of Neurodegenerative Disorders Montpellier France; ^9^ Assistance Publique‐Hôpitaux de Paris (AP‐HP) Département de Neurologie, Centre des Maladies Cognitives et Comportementales, GH Pitié‐Salpêtrière Paris France; ^10^ Université de Paris EA 4468, APHP, Hopital Broca, Memory Resource and Research Centre of de Paris‐Broca‐Ile de France Paris France; ^11^ Université de Strasbourg Hôpitaux Universitaires de Strasbourg, CM2R, pôle de Gériatrie, Laboratoire ICube, FMTS, CNRS, équipe IMIS Strasbourg France; ^12^ Université de Montpellier CHU Montpellier, Memory Research and Resources center of Montpellier, department of Neurology, Inserm INM NeuroPEPs team, excellence center of neurodegenerative disorders Montpellier France; ^13^ Univ. Lille, Inserm, CHU Lille, U1172‐LilNCog, LiCEND, LabEx DISTALZ Lille France; ^14^ Univ. Lille, Inserm U1171 Degenerative and Vascular Cognitive Disorders Lille France; ^15^ Université Paris‐Saclay APHP, Hôpital Paul Brousse, département de gériatrie, Équipe MOODS, Inserm 1178 Villejuif France; ^16^ Department of Gerontology Centre Hospitalier de la Côte Basque Bayonne France; ^17^ Department of clinical gerontology Université de Nantes, EA 4334 Movement‐Interactions‐Performance, CHU Nantes, Memory Research Resource Center of Nantes Nantes France; ^18^ Department of Gérontology 2 Sorbonne Université APHP Centre Hospitalier Dupuytren Draveil France; ^19^ Neurology Department CHU de Nimes, Hôpital Caremeau Nimes France; ^20^ Hôpital Léopold Bellan Service de Neuro‐Psycho‐Gériatrie Memory Clinic Paris France; ^21^ Department of Neurology Univ. Lille CHU de Lille, Memory Resource and Research Centre of Lille Lille France; ^22^ Université de Paris, APHP, Groupe Hospitalier Saint Louis‐LariboisièreFernand Widal, Center of Cognitive Neurology Paris France; ^23^ Université de Toulouse III, CHU La Grave‐Casselardit, Memory Resource and Research Centre of Midi‐Pyrénées Toulouse France; ^24^ Université de Lorraine, CHRU de Nancy, Memory Resource and Research Centre of Lorraine Vandoeuvre‐lès‐Nancy France; ^25^ Université de Paris, APHP, Hôpital europeen Georges Pompidou, Service de Gériatrie Paris France; ^26^ CHU de Poitiers, Memory Resource and Research Centre of Poitiers Poitiers France; ^27^ CHU Charles Nicolle, Memory Resource and Research Centre of HauteNormandie Rouen France; ^28^ Department of Gérontology 1 APHP, Centre Hospitalier Émile‐Roux Limeil‐Brévannes France; ^29^ CHU d'Amiens‐Picardie, Memory Resource and Research Centre of AmiensPicardie Amiens France; ^30^ Sorbonne Université, APHP, Hôpitaux Universitaires Pitie‐Salpêtrière‐Charles‐Foix, Service de Gériatrie Ambulatoire Paris France; ^31^ Université Côte d'Azur, CHU de Nice, Memory Research Resource Center of Nice, CoBTek lab Nice France; ^32^ CHU de Limoges, Memory Research Resource Center of Limoges Limoges France; ^33^ Department of Neurology and CNR‐MAJ Normandie Univ, UNIROUEN, Inserm U1245, CHU de Rouen Normandy Center for Genomic and Personalized Medicine, CIC‐CRB1404 Rouen France; ^34^ CHU de Besançon, Memory Resource and Research Centre of Besançon Franche‐Comté Besançon France; ^35^ Université de Reims Champagne‐Ardenne, EA 3797, CHU de Reims, Memory Resource and Research Centre of Champagne‐Ardenne Reims France; ^36^ CHU d'Angers, Memory Resource and Research Centre of Angers Angers France; ^37^ CHRU de Tours, Memory Resource and Research Centre of Tours Tours France; ^38^ Université de Strasbourg, CHRU de Strasbourg, Memory Resource and Research Centre of Strasbourg/Colmar, Inserm U‐118 Strasbourg France; ^39^ Department of Neurology Univ Paris Est Creteil, EA 4391 Excitabilité Nerveuse et Thérapeutique, CHU Henri Mondor Créteil France; ^40^ Department of Neurology Hôpital Fondation Rothschild Paris France; ^41^ Department of Gerontology CHU d'Amiens‐Picardie Amiens France; ^42^ Sorbonne Université, APHP, Hôpitaux Universitaires Pitie‐Salpêtrière‐Charles Foix, Memory Resource and Research Centre, Centre des Maladies Cognitives et Comportementales IM2A, Inserm UMR 8256 Paris France; ^43^ Université François‐Rabelais de Tours, CHRU de Tours, Memory Resource and Research Centre of Tours, Inserm CIC 1415 Tours France; ^44^ Sorbonne Université, APHP, Hôpitaux Universitaires Pitie‐Salpêtrière‐Charles Foix, Memory Resource and Research Centre, Centre des Maladies Cognitives et Comportementales IM2A Paris France; ^45^ Université Bourgogne Franche‐Comté, Laboratoire de Recherches Intégratives en Neurosciences et Psychologie Cognitive, CHU de Besançon, Memory Resource and Research Centre of Besançon Franche‐Comté Besançon France; ^46^ Université François‐Rabelais de Tours, CHRU de Tours, UMR Inserm U1253 Tours France; ^47^ Université de Limoges, EA 6310 HAVAE, CHU de Limoges, Memory Research Resource Center of Limoges Limoges France; ^48^ Université Paris‐Saclay, Neurospin, CEA, CNRS, cati‐neuroimaging.com, CATI Multicenter Neuroimaging Platform Gif‐sur‐Yvette France; ^49^ Université de Paris, Institute of Psychiatric and Neurosciences, Inserm UMR‐S 1266 Paris France; ^50^ Université de Montpellier, CHU Montpellier, LBPC, Inserm Montpellier France

**Keywords:** Alzheimer's disease, caffeine, CSF biomarkers, memory, mild cognitive impairment

## Abstract

**INTRODUCTION:**

We investigated the link between habitual caffeine intake with memory impairments and cerebrospinal fluid (CSF) biomarkers in mild cognitive impairment (MCI) and Alzheimer's disease (AD) patients.

**METHODS:**

MCI (*N* = 147) and AD (*N* = 116) patients of the Biomarker of AmyLoid pepTide and AlZheimer's diseAse Risk (BALTAZAR) cohort reported their caffeine intake at inclusion using a dedicated survey. Associations of caffeine consumption with memory impairments and CSF biomarkers (tau, p‐tau181, amyloid beta 1‐42 [Aβ_1‐42_], Aβ_1‐40_) were analyzed using logistic and analysis of covariance models.

**RESULTS:**

Adjusted on Apolipoprotein E (*APOE* ε4), age, sex, education level, and tobacco, lower caffeine consumption was associated with higher risk to be amnestic (OR: 2.49 [95% CI: 1.13 to 5.46]; *p* = 0.023) and lower CSF Aβ_1‐42_ (*p* = 0.047), Aβ_1‐42_/Aβ_1‐40_ (*p* = 0.040), and Aβ_1‐42_/p‐tau181 (*p* = 0.020) in the whole cohort.

**DISCUSSION:**

Data support the beneficial effect of caffeine consumption to memory impairments and CSF amyloid markers in MCI and AD patients.

**Highlights:**

- We studied the impact of caffeine consumption in the BALTAZAR cohort.
- Low caffeine intake is associated with higher risk of being amnestic in MCI/AD patients.
- Caffeine intake is associated with CSF biomarkers in AD patients.

## 1 BACKGROUND

Alzheimer's disease (AD) is characterized by a progressive cognitive decline linked to both extracellular deposits of aggregated amyloid beta (Aβ) peptides into plaques and the intraneuronal aggregation of hyperphosphorylated tau proteins.^1^ Besides aging, AD risk depends on various genetic and environmental factors.^2^, ^3^

Caffeine is the most widely consumed psychoactive agent worldwide via dietary intake from coffee, tea, or soda beverages.^4^ Coffee consumption has been inversely associated with total and cause‐specific mortality in a large prospective cohort of participants aged 50 to 71 years at baseline (National Institutes of Health—AARP Diet and Health Study) with an 8‐year follow‐up.^5^ Compelling evidence supports acute caffeine's ability to increase/improve wakefulness, alertness, and memory ([6]; for a review see Cunha^7^ and van Dam et al.^8^). Various longitudinal, cross‐sectional, and retrospective studies support the idea that habitual coffee/caffeine consumption reduces cognitive decline in the elderly^9^, ^10^, ^11^, ^12^, ^13^, ^14^, ^15^, ^16^, ^17^, ^18^ (for reviews see ref. Cunha^7^ and Yelanchezian et al.^19^). Further, other works suggest that coffee/caffeine intake reduces dementia or AD risk.^20^, ^21^, ^22^, ^23^ Caffeine consumption has also been associated with a decrease of behavioral symptoms in patients with dementia.^24^ All these observations, obtained mostly during follow‐up on non‐demented elderly populations, have been acknowledged in meta‐analysis studies.^25^, ^26^, ^27^, ^28^

Although experimental works using both amyloid and tau models show the beneficial impact of caffeine on the development of AD lesions^29^, ^30^, ^31^ (for a review see Cunha^7^ and Yelanchezian et al.^19^), it remains largely unclear whether caffeine consumption is associated with brain levels of amyloid and tau in MCI or AD individuals. Two studies support, in cognitively normal aged individuals, a significant association between lower coffee intake and a higher Aβ positivity, as seen by positron emission tomography (PET),^32^, ^33^ but these data have been discussed elsewhere.^34^ Notably, the link between caffeine intake and cerebrospinal fluid (CSF) biomarkers, including amyloid peptides Aβ and tau protein, in individuals presenting with MCI and AD has been largely overlooked. Only one study could not find an association of caffeine consumption, caffeine concentration in plasma, or caffeine concentration in the CSF with the levels of the core AD CSF biomarkers, in a cohort that included 88 patients with AD or mild cognitive impairment (MCI).^35^

In this context, the present study aimed to reinvestigate the link between habitual caffeine intake with CSF levels of amyloid Aβ40, Aβ42, tau, and p‐tau in the clinically defined Biomarker of AmyLoid pepTide and AlZheimer's diseAse Risk (BALTAZAR) cohort, including non‐amnestic MCI (naMCI), amnestic MCI (aMCI), and AD patients.

## 2 METHODS

### 2.1 Study population

This study is ancillary to BALTAZAR, a multicenter (23 memory centers) prospective cohort study (ClinicalTrials.gov, Identifier #NCT01315639) including participants with MCI and AD at baseline from September 2010 to April 2015 and with an ongoing 3‐year follow‐up. All participants or their legal guardians gave written informed consent. The study was approved by the Paris Ethics Committee (CPP Ile de France IV Saint Louis Hospital). All participants were Caucasian community dwellers and had caregivers. Inclusion criteria for AD participants were age ≥45 years, probable AD based on the *Diagnostic and Statistical Manual of Mental Disorders*, 4th Edition, Text Revision (DSM IV‐TR), and the National Institute of Neurological and Communicative Disorders and Stroke and Alzheimer's Disease and Related Disorders Association criteria^36^ and from mild to moderate stage (Mini‐Mental State Examination [MMSE] score ≥15). Inclusion criteria for MCI participants were age ≥70 years with MCI diagnostic criteria according to Petersen. ^37^ Exclusion criteria were non‐AD dementia (ie, vascular dementia, Lewy body or Parkinson's disease dementia, and frontotemporal dementia); genetic forms of AD; major depression according to DSM IV‐TR or geriatric depression scale > 20/30; other diseases that could interfere with cognitive evaluation; diseases with short‐term survival; use of cholinesterase inhibitors or methyl‐D‐aspartate receptor partial antagonists before inclusion (for participants with MCI); and illiterateness or less than 4 years of education.

At baseline, all the participants underwent clinical, neuropsychological, and biological assessments, and participants without contraindications underwent magnetic resonance imaging (MRI) brain examinations. Cognitive evaluations were performed by neuropsychologists after training programs to harmonize the evaluation. At inclusion, MCI and AD participants underwent a neuropsychological test battery that included global cognitive assessment with the MMSE (normal score 30/30), Clinical Dementia Rating (CDR) scale (CDR sum of boxes: normal score 0/18). Functional disability was assessed using instrumental activities of daily living (IADL, normal score 14/14) (for the description of all tests performed in the cohort see Hanon et al.^38^). MCI participants were then categorized as aMCI or naMCI according to the presence of memory impairment on the free and cued selective recall reminding test related to age, sex, and educational level.^39^ Conversion to dementia in the naMCI group was very low (2.5%) compared to the aMCI group (22.4%).

### 2.2 Habitual caffeine consumption

This study enrolled 263 patients (see flowchart in Figure S1) in which habitual caffeine intake was assessed using an in‐house validated self‐survey,^40^ completed together by the patients and their caregivers. At inclusion, the daily intake of caffeine‐containing items (coffee, tea, chocolate, sodas) was reported. The daily caffeine intake at inclusion was then calculated as milligrams per day. Participants were dichotomized according to their median caffeine consumption (216 mg/day), defining “low caffeine consumption” (≤216 mg/day) and “high caffeine consumption” (>216 mg/day) groups.

### 2.3 CSF and plasma biomarker measurements

Blood and CSF samples were collected at the same time. Investigators involved in the biological analysis were blinded to other assessments. A standard protocol was established beforehand and used throughout the study. CSF samples (>4 mL) were centrifuged (1000 × *g*, +4 C, 10 min) less than 4 h after collection using the same 10‐mL polypropylene tube (Catalogue No. #62.610.201; Sarstedt, Germany), aliquoted into polypropylene protein low‐binding tubes (LoBind microtube‐ref 022431064; Eppendorf), and stored at −80°C. CSF tau, p‐tau181, Aβ40, and Aβ42 were measured in duplicate using the same aliquot in a single centralized laboratory (IRMB, Montpellier, France) using commercially available ELISA kits (Euroimmun Aβ_1‐40_ and Aβ_1‐42_, Innotest htau and Innotest p‐tau181). It is noteworthy that about half of patients did not accept lumbar puncture, which explains the missing data on CSF biomarkers.

Regarding plasma, all centers used the same 10 mL collection tube with EDTA (BD Vacutainer K2E Catalogue No. 367,525, Becton Dickinson, Rungis, France). After centrifugation (2500 × *g*, 10 min), the supernatants were aliquoted into polypropylene protein low‐binding tubes (LoBind‐microtube, Ref #022431064; Eppendorf, Hamburg, Germany) and stored at −80°C. Plasma Aβ40 and Aβ42 peptide assay was performed in a single centralized laboratory (Inserm UMRS1172, Lille) using the INNO‐BIA kit (Fujirebio Europe NV, formerly Innogenetics NV, Belgium), based on a multiplex xMAP technique with a LABScan‐200 system (Luminex BV). Plasma p‐tau181 was determined using a commercial Simoa plasma p‐tau181 Advantage V1 kit, and plasma neurofilament light (NfL) using a commercial Simoa NF‐light Advantage Kit (Quanterix, Billerica, MA, USA) in a single centralized laboratory (IRMB, Montpellier, France). Internal quality controls (IQCs) represented by serum pool aliquots were used to monitor the accuracy of Simoa. All samples were measured after a single thaw.

**RESEARCH IN CONTEXT**

1. **Systematic review**: The authors reviewed the literature using traditional (eg, PubMed) sources, meeting abstracts, and presentations. While the impact of habitual caffeine consumption on age‐related cognitive decline has been addressed in longitudinal studies and meta‐analyses, its impact in mild cognitive impairment (MCI) and Alzheimer's disease (AD) patients remains ill defined, in particular regarding cerebrospinal fluid (CSF) biomarkers.
2. **Interpretation**: Our data support an association of lower caffeine consumption with a higher risk of being amnestic as well as with deleterious changes in CSF biomarkers of MCI and AD patients.
3. **Future directions**: The article proposes a framework for future interventional clinical studies aimed at evaluating the effect of caffeine on clinical progression and biomarker changes during the course of AD and at better understanding the associated biological mechanisms.

### 2.4 Statistical analysis

Categorical variables are reported as frequency (percentage). Continuous variables are reported as mean (standard deviation [SD]) in the case of normal distribution or median (interquartile range [IQR]) otherwise. Normality of distributions was assessed using histograms and the Shapiro–Wilk test.

Patient characteristics and CSF biomarkers were compared between the three diagnostic groups (naMCI, aMCI, and AD) using chi‐squared test (or Fisher's exact test in the case of an expected value <5) for categorical variables and using analysis of variance or Kruskal–Wallis test for quantitative variables. CSF data were not imputed as the proportion of missing data was too important and not random. For MCI patients, association between biomarkers and conversion to AD were evaluated using a Cox proportional hazards model. The proportional hazards assumption and the log‐linearity assumption for quantitative variables were assessed by examining the scaled Schoenfeld residuals plots and the Martingale residual plots. Hazard ratios were estimated with their 95% confidence interval (CI).

The impact of low or high caffeine consumption on the diagnostic group was evaluated using a multinomial logistic regression model adjusted on predefined confounding factors (Apolipoprotein E [*APOE* ε4], age, sex, education level, and tobacco consumption). Odds ratios (ORs) were derived from models with their 95% CIs. The same analysis was performed by regrouping aMCI and DA groups using a logistic regression model adjusted on predefined confounding factors.

The impact of low or high caffeine consumption on biomarkers was evaluated using an analysis of covariance (ANCOVA) adjusted on predefined confounding factors and after applying a log transformation of the CSF biomarkers and plasma NfL levels. Mean difference was derived from models with their 95% CIs.

All statistical tests were done at the two‐tailed *α*‐level of 0.05 using SAS software version 9.4 (SAS Institute, Cary, NC, USA).

## 3 RESULTS

### 3.1 Demographic and clinical characteristics at baseline

Two hundred and sixty‐three participants of the BALTAZAR cohort with available caffeine data were included (40 naMCI, 107 aMCI, and 116 AD; Table 1). At baseline, among the 263 participants, 38.0% (*N* = 100) were males, 40.8% (*N* = 106) had at least a high school diploma, and 74.8% (*N* = 193) never smoked. The median MMSE score was 26 (IQR: 23 to 28) and 47.7% (*N* = 114) were *APOE* ε4 carriers. The median caffeine consumption was 216 (IQR: 84 to 374) mg/day. During the clinical follow‐up period (6 to 36 months), 24 MCI participants developed dementia and converted to probable AD. Analysis of CSF biomarkers at inclusion showed, as expected, higher CSF total tau (tau) and phospho‐tau (p‐tau181) levels and lower amyloid Aβ42 levels, Aβ42/Aβ40, and Aβ42/p‐tau181 ratios in AD patients compared to naMCI and aMCI individuals. As shown in Table S1, we also described CSF and plasma biomarker changes in the MCI patients converting to dementia (mainly AD) versus non‐converters: as in the whole BALTAZAR MCI cohort,^41^ we found higher CSF tau and p‐tau181 levels; lower CSF Aβ42 levels and Aβ42/Aβ40 and Aβ42/p‐tau181 ratios, and lower plasma Aβ42/Aβ40. Similarly, hippocampal volume, MMSE, and IADL were significantly lower, while CDR was higher in AD versus aMCI and naMCI (Table 1).

**TABLE 1:** General characteristics, global cognitive assessment, and CSF biomarkers at baseline.

	*N*	Overall *N* = 263	*N*	naMCI *N* = 40	*N*	aMCI *N* = 107	*N*	AD *N* = 116	*p*‐value
**Age (years)**	263	77 ± 6	40	76 ± 5	107	77 ± 5	116	78 ± 7	0.11
**Male (*N*, %)**	263	100 (38.0)	40	9 (22.5)	107	50 (46.7)	116	41 (35.3)	0.019
**Education level (*N*, %)**	260		38		106		116		0.038
Primary		47 (18.1)		8 (21.1)		13 (12.1)		26 (22.6)	
Secondary		107 (41.2)		10 (26.3)		45 (42.1)		52 (45.2)	
High school diploma or above		106 (40.8)		20 (52.6)		49 (45.8)		37 (32.2)	
**Tobacco (*N*, %)**	258		38		105		115		NA
Never		193 (74.8)		29 (76.3)		73 (69.5)		91 (79.1)	
Current		7 (2.7)		2 (5.3)		3 (2.9)		2 (1.7)	
Former		58 (22.5)		7 (18.4)		29 (27.6)		22 (19.1)	
**BMI (kg/m^2^)**	262	25.2 ± 3.6	40	24.5 ± 3.2	106	24.9 ± 3.3	116	25.8 ± 3.9	0.058
**Caffeine (mg/day)**	263	216 (84 to 374)	40	266 (182 to 427)	107	197 (79 to 332)	116	216 (76 to 385)	0.16
Low‐caffeine group (≤216 mg/day)		132 (50.2)		15 (37.5)		59 (55.1)		58 (50.0)	
High‐caffeine group (>216 mg/day)		131 (49.8)		25 (62.5)		48 (44.9)		58 (50.0)	
**Comorbidity (*N*, %)**									
Hypertension	261	190 (72.8)	38	28 (70.0)	107	82 (76.6)	116	80 (70.2)	0.51
Mellitus diabetes	238	35 (14.7)	38	4 (10.5)	99	14 (14.1)	101	17 (16.8)	0.63
Dyslipidemia	260	106 (40.8)	39	20 (50.0)	105	45 (42.5)	116	41 (36.0)	0.27
History of stroke or TIA	262	18 (6.9)	39	0 (0.0)	107	9 (8.5)	116	9 (7.8)	0.16
History of depression	261	59 (22.6)	38	11 (27.5)	107	25 (23.8)	116	23 (19.8)	0.56
**Relative hippocampal volume**	229	0.33 ± 0.09	35	0.40 ± 0.05	91	0.35 ± 0.08	103	0.29 ± 0.09	<0.001
*APOE* ε4 carrier (***N*, %)**	239	114 (47.7)	40	12 (30.0)	105	50 (47.6)	94	52 (55.3)	0.027
**Global cognitive assessment**									
CDR sum of boxes (/18)	263	2.0 (0.5 to 4.0)	40	0.5 (0.5 to 0.6)	107	1.0 (0.5 to 2.0)	116	4.3 (2.5 to 5.6)	<0.001
MMSE (/30)	252	26 (23 to 28)	37	28 (27 to 30)	105	27 (25 to 28)	110	23 (20 to 25)	<0.001
IADL score (/14)	257	13 (11 to 14)	39	14 (14 to 14)	105	14 (12 to 14)	113	11 (7 to 13)	<0.001
**CSF biomarkers**									
Aβ40 (pg/mL)	107	6963 (5925 to 8647)	18	6801 (6293.0 to 9547)	49	7350 (5925 to 9023)	40	6799 (58278 to 8182)	0.48
Aβ42 (pg/mL)	107	589 (411 to 956)	18	853 (673 to 1030)	49	667 (431 to 1183)	40	471 (403 to 624)	<0.001
Aβ42/Aβ40 (%)	107	8.2 (6.7 to 13.2)	18	11.2 (8.3 to 14.3)	49	9.3 (6.7 to 15.6)	40	7.3 (6.1 to 8.7)	<0.001
tau (pg/mL)	118	399 (309 to 625)	18	311 (214 to 410)	56	371 (280 to 600)	44	596 (392 to 710)	<0.001
p‐tau181 (pg/mL)	120	61 (48 to 82)	18	52 (45 to 60)	57	56 (48 to 77)	45	69 (63 to 98)	<0.001
Aβ42/p‐tau	103	9.2 (5.7 to 19.3)	18	15.5 (11.6 to 22.8)	47	11.5 (6.5 to 21.9)	38	7.0 (4.8 to 9.5)	<0.001

*Note*: Values are presented as mean ± SD or median (interquartile range) for quantitative variables and as frequency (percentage) for categorical variables.

Abbreviations: AD, Alzheimer's disease; aMCI: amnestic mild cognitive impairment; BMI, body mass index; CDR SOB, Clinical Dementia Rating Sum of Boxes; CSF, cerebrospinal fluid; IADL, instrumental activities of daily living; MMSE, Mini‐Mental State Examination; naMCI, non‐amnestic mild cognitive impairment; *N*, number of available observations; TIA, transient ischemic attack.

### 3.2 Association of caffeine consumption with the diagnostic groups at baseline according to the cognitive status (AD, aMCI, naMCI)

Participants were dichotomized according to their median caffeine consumption (216 mg/day) in the overall cohort as well as in each subgroup (AD, aMCI, naMCI). Body mass index (BMI) was not significantly different between low and high caffeine consumers (mean BMI: 25.3 ± 3.8 in the low vs 25.2 ± 3.5 in the high consumer group; *p* = .93, Student's *t* test). At baseline, we did not find a statistical difference in caffeine intake between naMCI, aMCI, and AD patients (Table 1), suggesting that the development of AD is not associated with a change in consumption, at least versus MCI patients. Also, the median caffeine consumption calculated for MCI patient that convert (199.5 mg/day) or not (217.7 mg/day) was not found significantly different (*p* = 0.88). However, after adjustment on *APOE* ε4, age, sex, education level, and tobacco consumption, and using the naMCI group as reference, we found a significant association of a lower caffeine consumption with a higher risk to be aMCI (OR: 2.72 [95% CI: 1.17 to 6.30]) and a similar effect size, even non‐significant, for higher risk of being AD (OR:2.31 [95% CI: 0.98 to 5.40]; Table 2). When aMCI and AD groups were combined, the association of a lower caffeine consumption with higher risk of being amnestic was significant (OR: 2.49 [95% CI: 1.13 to 5.46]; *p* = 0.023) (Table 2). Nevertheless, a Kaplan–Meyer analysis indicated that the rate of MCI conversion to AD was not modified regarding the caffeine consumption class (*p* = 0.94; not shown).

**TABLE 2:** Association of caffeine consumption according to cognitive status (naMCI, aMCI, AD).

Overall	*N* = 263	Low caffeine consumption *N* = 132	High caffeine consumption *N* = 131	Odds ratio (95% CI)	*p*‐value
**naMCI_aMCI_AD (*N*, %)**					0.062
naMCI	40 (15.2)	15 (11.4)	25 (19.1)	1.00 (réf.)	–
aMCI	107 (40.7)	59 (44.7)	48 (36.6)	2.72 (1.17 to 6.30)	**0.019**
AD	116 (44.1)	58 (43.9)	58 (44.3)	2.31 (0.98 to 5.40)	0.054
**naMCI_aMCI+AD (*N*, %)**					
naMCI	40 (15.2)	15 (11.4)	25 (19.1)	1.00 (réf.)	–
aMCI+AD	223 (84.8)	117 (88.6)	106 (80.9)	2.49 (1.13 to 5.46)	**0.023**

*Note*: Values are presented as frequency (percentage). Odds ratios and *p* values were adjusted for *APOE* ε4, age, sex, education level, and tobacco consumption.

Abbreviations: AD, Alzheimer's disease; aMCI, amnestic mild cognitive impairment; CI, confidence interval; naMCI, non‐amnestic mild cognitive impairment; *N*, number of available observations.

### 3.3 Association of caffeine consumption with CSF and plasma biomarkers at baseline

Among the whole cohort, after adjustment on *APOE* ε4, age, sex, education level, and tobacco consumption, lower caffeine consumption was found to be associated with lower CSF Aβ42 levels (*p* = 0.047) as well as lower Aβ42/Aβ40 (*p* = 0.040) and Aβ42/p‐tau181 ratio (*p* = 0.020; Table 3). No significant difference was observed regarding CSF total tau and p‐tau181 levels based on caffeine consumption (Table 3). We also evaluated plasma biomarkers (p‐tau181, Aβ42, Aβ40, and NfL) but did not observe significant differences per the caffeine consumption group (Table S2).

**TABLE 3:** Association of caffeine consumption with CSF biomarkers at baseline.

CSF biomarkers	*N*	Low caffeine consumption *N* = 132	*N*	High caffeine consumption *N* = 131	Mean difference (95% CI)	*p*‐value
tau (pg/mL)	55	400 (311 to 627)	63	385 (287 to 622)	0.14 (−0.06 to 0.35)	0.17
p‐tau181 (pg/mL)	56	62 (49 to 84)	64	60 (47 to 80)	0.13 (−0.03 to 0.30)	0.11
Aβ42 (pg/mL)	50	577 (411 to 953)	57	658 (431 to 956)	−0.21 (−0.41 to 0.00)	**0.040**
Aβ40 (pg/mL)	50	7075 (5934 to 8647)	57	6795 (5874 to 9007)	−0.05 (−0.18 to 0.09)	0.47
Aβ42/Aβ40 (%)	50	8.1 (6.7 to 14.0)	57	8.4 (6.8 to 12.9)	−0.17 (−0.32 to −0.01)	**0.047**
CSF Aβ42/p‐tau	49	8.5 (5.7 to 17.2)	54	10.5 (6.5 to 21.3)	−0.34 (−0.61 to −0.06)	**0.020**

*Note*: Values are presented as median (IQR). Mean differences and *p* values were calculated on log transformed data and were adjusted for *APOE* ε4, age, sex, education level, and tobacco consumption, using high caffeine consumption as reference.

Abbreviations: CI, confidence interval; CSF, cerebrospinal fluid; *N*, number of available observations.

## 4 DISCUSSION

In this study, we evaluated the impact of caffeine consumption on clinical outcomes and biomarkers at baseline in the BALTAZAR cohort, including MCI and AD patients. Our data show an association of a lower caffeine consumption with memory disorders related to AD and aMCI at inclusion. Importantly, our data also demonstrate that caffeine is associated with changes in CSF biomarkers of AD patients, particularly with regard to the amyloid component.

The association fits well with few, and potentially underpowered, previous retrospective studies investigating the impact of caffeine consumption on populations of patients presenting with MCI or AD. Maia and de Mendonça^21^ showed a significantly lower average consumption of caffeine (74 mg/day, *N* = 54) during the 20 years preceding AD development in subjects as compared with age‐matched non‐demented subjects (approximately 200 mg/day, *N* = 54). In the same way, Cao et al.^42^ suggested that plasma caffeine concentrations were approximately 50% lower in subjects converting to dementia (*N* = 15) (presumably comparable to our aMCI group at high risk of converting to dementia) versus subjects that did not convert (*N* = 9) (presumably comparable to our naMCI group). Interestingly, one study,^43^ in line with experimental data,^44^ found that higher caffeine intake was associated with better function in overall cognition, encompassing episodic memory, executive function, semantic categorization, and working memory, in a cohort of approximately 600 subjects presenting with diabetes, known to be a significant risk factor for dementia,^45^ as are aMCI individuals.^46^ These data support a benefit of habitual caffeine consumption in at‐risk individuals that would warrant extending more broadly cognitive endophenotypes at MCI inclusion and follow‐up regarding caffeine consumption. Besides an impact on brain lesions (see following discussion), association between caffeine consumption and memory might primarily relate to the ability of caffeine to act on synaptic plasticity. Indeed, in rodents, caffeine's effect on hippocampal slices is to enhance basal synaptic transmission, long‐term potentiation (LTP), and sharp wave–ripple complexes, which underlie memory consolidation.^47^, ^48^, ^49^, ^50^ Caffeine also controls neuronal excitability and LTP‐like effects in the human cortex.^51^, ^52^ More recently, we also demonstrated that regular caffeine intake acted on epigenomic and transcriptional processes that would improve the signal‐to‐noise ratio during information encoding, favoring the functioning of circuits involved in learning.^53^ The beneficial effect of caffeine on synaptic plasticity during MCI and AD would be particularly ascribed to its ability to block adenosine receptors,^4^, ^7^ particularly the A_2A_R subtypes.^54^

Importantly, we showed a significant association across the BALTAZAR cohort between lower caffeine intake and lower CSF levels of Aβ42 as well as Aβ42/Aβ40 and Aβ42/p‐tau181 ratios. To our knowledge, this is the first study to report an association between caffeine and CSF AD biomarkers. Only one previous study addressed, unsuccessfully, such an association in another population.^35^ However, this study included a more limited number of subjects (37 MCI and 51 AD), the biomarker assay techniques were different, and, for an unclear reason, the median caffeine consumption was two times lower (around 100 mg/day) than in our population. Using our in‐house survey – developed to obtain more precise information on consumption habits than caffeine unit or mugs – and considering all types of caffeine consumption and volume ingested, we estimated the median consumption of our population to be 216 mg/day. This amount was in line with previous studies in cohorts of a similar age^18^, ^32^, ^43^ and the average consumption in French population as shown by Fredholm et al.^4^ Our CSF data point to a particular impact of caffeine on amyloid burden. This is in accordance with a Korean study^33^ that included elderly subjects (mean age 71 years old) without dementia (282 cognitively normal and 129 with MCI), showing an association between coffee consumption greater than two cups/day (ie, about 200 mg) with amyloid Pittsburgh compound B (PIB)‐PET positivity. Another recent study involving only cognitively normal subjects (mean age 71 years) also found a significant association between higher caffeine consumption and slower accumulation of cerebral amyloid load measured by PIB‐PET during a 126‐month follow‐up.^32^

We also evaluated plasma biomarkers, except plasma total tau (t‐tau) levels, as it has been acknowledged that they do not provide valuable information regarding tau brain levels. Indeed, plasma t‐tau concentrations do not to correlate with CSF t‐tau or PET and neuropathological evidence of AD.^55^, ^56^, ^57^, ^58^ Evaluation of plasma p‐tau181 and Aβ did not reveal differences in connection with caffeine consumption. Such a lack of a difference could be explained by the smaller variations observed in amyloid and p‐tau levels in plasma compared to CSF. Indeed, in the BALTAZAR cohort, we previously observed an association between CSF biomarkers and plasma amyloid levels as well as plasma p‐tau.^38^, ^59^ However, the difference or lack thereof in mean levels between MCI converters was respectively for plasma and CSF: −2.1 and −283 pg/mL for Aβ_1‐42_ and +0.9 and + 21.7 pg/mL for p‐tau181.^41^, ^59^ Therefore, the lack of difference in the present study could be simply due to a lack of statistical power linked to an insufficient number of patients with known caffeine consumption. Alternatively, this could also suggest that caffeine mainly has a direct central effect on brain processes rather an indirect effect through peripheral inflammation, amyloid clearance, or metabolism. Regarding plasma NfL levels, also not associated with caffeine consumption, we previously showed in the BALTAZAR cohort that they were not linked to conversion to dementia.^60^ Therefore, the observed variations of CSF amyloid levels based on caffeine consumption in our study are compatible with the lack of plasma NfL variations. NfL is well known as a non‐specific biomarker for neurodegeneration.^61^ The lack of difference observed for plasma NfL also therefore suggests a lack of caffeine protection on a terminal neurodegenerative process but an upstream effect on amyloidogenic pathway and/or neuronal plasticity.

Several mechanisms might indeed explain the effect of caffeine on amyloid pathology revealed by our CSF biomarker results. In vitro data by Sharma et al.^62^ showed that the hydrophobic core‐recognition motif of amyloid formation was physically blocked by caffeine, thereby abolishing self‐assembly formation. Also, Janitschke et al.^63^ showed that caffeine was able to decrease Aβ levels by shifting the amyloid precursor protein (APP) processing from the Aβ‐producing amyloidogenic to the non‐amyloidogenic pathway. Accordingly, several studies showed that amyloid mice treated with chronic caffeine administration had lower hippocampal Aβ levels associated with a reduced presenilin 1 (PS1) and β‐secretase (BACE1) levels.^29^, ^30^, ^64^ These observations are likely in line with the ability of the A_2A_ receptor, an important pharmacological target of caffeine, to modulate Aβ production in vitro^65^ and in vivo.^66^ Further, in addition to acting on processes driving amyloid peptide formation and/or aggregation, caffeine could also enhance brain amyloid clearance in C57Bl6/J mice.^67^ In agreement with this, as hypothesized, caffeine would be likely to increase the production of CSF,^68^ enhancing CSF turnover and, subsequently, presumably improving Aβ brain clearance (for a review see Mehta and Mehta^69^). This attractive hypothesis, however, awaits definitive verification. Non‐mutually exclusively, the effect of caffeine on amyloid load might also rely on its ability to restrain neuroinflammatory processes, which are known to impair the clearance of amyloid lesions by glial cells (see Launay et al.^70^ and Hansen et al.^71^ for reviews).

Puzzlingly, caffeine does not seem to impact CSF tau and p‐tau. The underlying reasons remain unclear. Actually, in sharp contrast to amyloid model studies, only a few works tried to link caffeine to tau. In a previous in vitro study,^72^ caffeine was shown to block the cell cycle at the G1 phase in neuroblastoma cells and lead to a decrease in tau phosphorylation. But the model used might not be relevant to AD. Only one study by our team demonstrated the beneficial impact of caffeine in a mouse model of AD‐like tauopathy.^31^ In that work, we showed that caffeine improved memory, slightly decreasing hippocampal p‐tau, without any effect on tau aggregation, but showing rather a strong impact on tau truncation. However, another study performed in a diabetes model showing increased p‐tau supported the notion that caffeine exacerbates tau hyperphosphorylation by promoting hypothermia.^73^ Therefore, the link between caffeine and tau remains unclear and warrants further investigation.

Overall, the strength of the present study is that it allows for the possibility of addressing the impact of habitual caffeine consumption in a cohort including only MCI and AD patients very well described at the clinical, neuropsychological, MRI, and CSF biomarker levels, as well as for the possibility of adjusting statistical analyses of known confounding factors for AD (*APOE* ε4, age, sex, education level) and caffeine intake (smoking). However, our study had several limitations. First, we used a survey previously used in patients with Huntington's disease (HD). As indicated in our initial paper,^40^ the reliability of the survey was assessed by a retest and found to be excellent. However, that survey was not re‐validated in the present population of MCI and AD patients. Nevertheless, it is particularly noteworthy that the survey was completed by both patients and caregivers, minimizing the risk of error. We are therefore confident of the reliability of our survey, since coffee intake is less prone to being misreported due to its long‐term habitual nature.

Notably, the median consumption measured in the BALTAZAR cohort (216 mg/day) is very close to what we that measured in our previous HD study (median 190 mg/day) as well as in another ongoing yet unpublished study on patients diagnosed with amyotrophic lateral sclerosis (median 250 mg/day; not shown). One limit of the present study is, however, that we only estimated the intake at inclusion and did not determine consumption of the previous 10 or 20 years, but our consumption data fit with the reported habitual caffeine consumption in the overall French population by Fredholm et al. (239 mg/day^4^). This supports the idea that, despite our survey being only used to quantify caffeine consumption at inclusion, the values may very well reflect long‐lasting habitual caffeine consumption. This question is, however, relevant since patients may radically change their lifestyles with the diagnosis of life‐threatening diseases but also because caffeine‐associated neuroprotection may result from long‐term caffeine intake rather than from the actual caffeine intake. In a retrospective case‐control study by Maia and Mendonca^21^ in Portuguese individuals, daily caffeine consumption was calculated for the period from early adulthood (age 25 years old) to 20 years before diagnosis of AD, but also for the period after the diagnosis of AD up to the time of the survey at inclusion. In this study, patients with AD had an average daily caffeine intake during the 20 years that preceded diagnosis of AD of 73.9 ± 97.9 mg, whereas the controls had an average daily caffeine intake of 198.7 ± 135.7 mg during the corresponding 20 years of their lifetime, the latter amount being actually comparable to our patients’ consumption levels. During the period from young adulthood (25 years old) to 20 years before diagnosis, in Maia's study, AD patients already had a lower average daily caffeine intake. The average daily caffeine intake further declined in AD patients after the diagnosis, reaching a value of 36.3 ± 64.1 mg, while matched controls remained stable.

In another study by Cao et al.,^42^ MCI patients converting to AD exhibited lower plasma caffeine levels compared with non‐converter MCI patients. Therefore, differences in caffeine consumption before the onset of any memory decline may in fact exist. Nevertheless, our current data do not fit at all with these observations. Indeed, at baseline, we did not find a statistical difference in caffeine intake between naMCI, aMCI, and AD patients, suggesting that the development of AD is not associated with a change in consumption, at least when compared with MCI patients. Further, the median caffeine consumption calculated for MCI patients that convert to AD (199.5 mg/day) or not (217.7 mg/day) was similar (*p* = 0.88). Therefore, in our cohort, it seems that we did not observe a drop in caffeine consumption linked to the development of memory deficits or AD. Our results are rather in line with the study of Solfrizzi and al.,^14^ where no significant variations of caffeine consumption could be observed in MCI patients during a mean follow‐up of 3 years.

The BALTAZAR cohort is a prospective study, and although the existing literature mostly evaluates the impact of caffeine at baseline,^19^ evaluating cognition over 3 years from inclusion of MCI and AD patients would have been essential to estimate the potential consumption changes versus cognitive decline as suggested by Solfrizzi et al.^14^ The lack of consumption data during follow‐up visits in the present multicentric study precludes performing an association study between caffeine intake and decline without bias. This is another limit, and prospective studies on cognition, biomarkers, and pathological evolution in MCI and AD patients should be carefully paired with a longitudinal evaluation of caffeine consumption. Finally, while in our study the sample size was larger than in the previous study of Travasos et al.,^35^ it remained limited, in particular regarding the naMCI group. Another limitation of our cross‐sectional study is that the associations observed did not allow us to establish with certainty a cause‐and‐effect relationship between caffeine consumption and the effects observed. Causality can only be demonstrated by a randomized double‐blind interventional trial comparing patients treated with a caffeine arm or a placebo. This is the reason why we set up the interventional Effect of CAFfeine on Cognition in Alzheimer's Disease clinical phase 3 trial (NCT04570085), which is currently recruiting.

[1] Université de Paris, EA 4468, APHP, Hopital Broca, Memory Resource and Research Centre of de Paris‐Broca‐Ile de France, F‐75013 Paris, France.
[2] Université de Strasbourg, Hôpitaux Universitaires de Strasbourg, CM2R, pôle de Gériatrie, Laboratoire ICube, FMTS, CNRS, équipe IMIS, F‐67000 Strasbourg, France.
[3] Université de Montpellier, CHU Montpellier, Memory Research and Resources center of Montpellier, department of Neurology, Inserm INM NeuroPEPs team, excellence center of neurodegenerative disorders, F‐34000 Montpellier, France.
[4] Univ. Lille, Inserm, CHU Lille, U1172‐LilNCog, LiCEND, LabEx DISTALZ, F‐59000 Lille, France.
[5] Univ. Lille, Inserm U1171 Degenerative and Vascular Cognitive Disorders, F‐59000 Lille, France.
[6] Université Paris‐Saclay, APHP, Hôpital Paul Brousse, département de gériatrie, Équipe MOODS, Inserm 1178, F‐94800 Villejuif, France.
[7] Centre Hospitalier de la Côte Basque, Department of Gerontology, F‐64100 Bayonne, France.
[8] Université de Nantes, EA 4334 Movement‐Interactions‐Performance, CHU Nantes, Memory Research Resource Center of Nantes, Department of clinical gerontology, F‐44000 Nantes, France.
[9] Sorbonne Université, APHP, Centre Hospitalier Dupuytren, Department of Gérontology 2, F‐91210 Draveil, France.
[10] CHU de Nimes, Hôpital Caremeau, Neurology Department, F‐30029 Nimes, France.
[11] Hôpital Léopold Bellan, Service de Neuro‐Psycho‐Gériatrie, Memory Clinic, F‐75014 Paris, France.
[12] Univ. Lille, CHU de Lille, Memory Resource and Research Centre of Lille, Department of Neurology, F‐59000 Lille, France.
[13] Université de Paris, APHP, Groupe Hospitalier Saint Louis‐LariboisièreFernand Widal, Center of Cognitive Neurology, F‐75010 Paris, France.
[14] Université de Toulouse III, CHU La Grave‐Casselardit, Memory Resource and Research Centre of Midi‐Pyrénées, F‐31300 Toulouse, France.
[15] Université de Lorraine, CHRU de Nancy, Memory Resource and Research Centre of Lorraine, F‐54500 Vandoeuvre‐lès‐Nancy, France.
[16] Université de Paris, APHP, Hôpital europeen Georges Pompidou, Service de Gériatrie, F‐75015, Paris, France.
[17] CHU de Poitiers, Memory Resource and Research Centre of Poitiers, F‐86000 Poitiers, France.
[18] CHU Charles Nicolle, Memory Resource and Research Centre of HauteNormandie, F‐76000 Rouen, France.
[19] APHP, Centre Hospitalier Émile‐Roux, Department of Gérontology 1, F‐94450 Limeil‐Brévannes, France.
[20] CHU d'Amiens‐Picardie, Memory Resource and Research Centre of AmiensPicardie, F‐80000 Amiens, France.
[21] Sorbonne Université, APHP, Hôpitaux Universitaires Pitie‐Salpêtrière‐Charles‐Foix, Service de Gériatrie Ambulatoire, F‐75013 Paris, France.
[22] Université Côte d'Azur, CHU de Nice, Memory Research Resource Center of Nice, CoBTek lab, F‐06100 Nice, France.
[23] CHU de Limoges, Memory Research Resource Center of Limoges, F‐87000 Limoges, France.
[24] Normandie Univ, UNIROUEN, Inserm U1245, CHU de Rouen, Department of Neurology and CNR‐MAJ, Normandy Center for Genomic and Personalized Medicine, CIC‐CRB1404, F‐76000, Rouen, France.
[25] CHU de Besançon, Memory Resource and Research Centre of Besançon Franche‐Comté, F‐25000 Besançon, France.
[26] Université de Reims Champagne‐Ardenne, EA 3797, CHU de Reims, Memory Resource and Research Centre of Champagne‐Ardenne, F‐51100 Reims, France.
[27] CHU d'Angers, Memory Resource and Research Centre of Angers, F‐49000 Angers, France.
[28] CHRU de Tours, Memory Resource and Research Centre of Tours, F‐37000 Tours, France.
[29] Université de Strasbourg, CHRU de Strasbourg, Memory Resource and Research Centre of Strasbourg/Colmar, Inserm U‐118, F‐67000 Strasbourg, France.
[30] Univ Paris Est Creteil, EA 4391 Excitabilité Nerveuse et Thérapeutique, CHU Henri Mondor, Department of Neurology, F‐94000 Créteil, France.
[31] Hôpital Fondation Rothschild, Department of Neurology, F‐75019 Paris, France.
[32] CHU d'Amiens‐Picardie, Department of Gerontology, F‐80000 Amiens, France.
[33] Sorbonne Université, APHP, Hôpitaux Universitaires Pitie‐Salpêtrière‐Charles Foix, Memory Resource and Research Centre, Centre des Maladies Cognitives et Comportementales IM2A, Inserm UMR 8256, F‐75013 Paris, France.
[34] Université François‐Rabelais de Tours, CHRU de Tours, Memory Resource and Research Centre of Tours, Inserm CIC 1415, F‐37000 Tours, France.
[35] Sorbonne Université, APHP, Hôpitaux Universitaires Pitie‐Salpêtrière‐Charles Foix, Memory Resource and Research Centre, Centre des Maladies Cognitives et Comportementales IM2A, F‐75013 Paris, France.
[36] Université Bourgogne Franche‐Comté, Laboratoire de Recherches Intégratives en Neurosciences et Psychologie Cognitive, CHU de Besançon, Memory Resource and Research Centre of Besançon Franche‐Comté, F‐25000 Besançon, France.
[37] Université François‐Rabelais de Tours, CHRU de Tours, UMR Inserm U1253, F‐37000 Tours, France.
[38] Université de Limoges, EA 6310 HAVAE, CHU de Limoges, Memory Research Resource Center of Limoges, F‐87000 Limoges, France.
[39] Université Paris‐Saclay, Neurospin, CEA, CNRS, cati‐neuroimaging.com, CATI Multicenter Neuroimaging Platform, F‐91190 Gif‐sur‐Yvette, France.
[40] Université de Paris, Institute of Psychiatric and Neurosciences, Inserm UMR‐S 1266, F‐75014 Paris, France.
[41] Université de Montpellier, CHU Montpellier, LBPC, Inserm, F‐34000 Montpellier, France.

## CONFLICT OF INTEREST STATEMENT

David Blum DB is a (non‐appointed) member of the scientific advisory board of Marvel Biosciences Corp. developing an A_2A_R antagonist but has no conflict of interest regarding the present work. Olivier Hanon received personal payment from Bayer, Servier, AstraZeneca, Boston Scientific, Vifor, BMS, Boehringer‐Ingelheim, and Pfizer for lectures and/or consulting services. Jean Sebastien Vidal received payment from Bayer for lectures made to a non‐profit medical association. Sylvain Lehmann received for his institution support from the following: H2020 MARIE SKŁODOWSKA‐CURIE “MIRIADE Multi‐omics Interdisciplinary Research Integration to Address DEmentia diagnosis,” ANR Flash Covid: “ProteoCOVID: Clinical proteomic characterization of the SARS‐CoV‐2 Spike protein to optimize its detection and the development of serological assays,” ANR “Silk_road: The Stable Isotope Labeling Kinetics (SILK) road to investigate human protein turnover in blood and cerebrospinal fluid,” EUROMET EMPIR “NeuroMet2 project: Metrology and Innovation for early diagnosis and accurate stratification of patients with neurodegenerative diseases.” During the past 36 months, he had a patent issued for “Procédé de préparation d'un échantillon peptidique” Brevet INPI n°1905247 du 20/05/2019 du CHU DE MONTPELLIER, UNIVERSITÉ DE MONTPELLIER and SPOT TO LAB. He received personal payment for participating on the Roche Diagnostic board on CSF biomarkers. Stéphanie Bombois, Bernadette Allinquant, Christiane Baret‐Rose, Jean‐Marc Tréluyer, Hendy Abdoul, Patrick Gelé, Christine Delmaire, Jean‐François Mangin, and Evelyne Galbrun have no conflicts of interest. Fredéric Blanc received honoraria from Roche and Biogen for presentations. He received payment to his institution as the national coordinator for the clinical trial DELPHIA for patients with dementia with Lewy bodies (Eisai). He received payment to his institution as the national coordinator for the clinical trial GRADUATE for patients with AD. Luc Buée received support for the present manuscript from LabEx DISTALZ. He received grants or contracts from the French National Research Agency (ANR) Fondation pour la Recherche Médicale (FRM). In the past 36 months, he had a patent on anti‐tau therapy issued. Jacques Touchon received payment or honoraria as chairman of CTAD. He received contracts from Regenlife and consulting fees from Regenlife. He is chairman of JT Conseil society. Jacques Hugon received grants or contracts from Protekt Therapeutics and consulting fees from Protekt Therapeutics. He is principal investigator of RECAGE project European Union H20/20 program and he is member of the scientific board of Fondation Philippe Chatrier, Paris, France. Bruno Vellas received grants or contracts from Biogen, Roche, and Lilly; consulting fees from Roche, Lily, Biogen, and Cerellis; and is part of WHO's ICOPE program (unpaid position). AthanBase Benetos is the president of the European Geriatric Medicine Society (unpaid position). He received support for attending meetings and/or travel from Fukuda company, for the Congress of the European Society of Hypertension, and received royalties or licenses from Cambridge University Editions. Gilles Berrut received a grant from Boehringer Ingelheim and consulting fees from Boehringer Ingelheim, Smart macadam Institut, bien vieillir Korian. Elena Paillaud has no conflicts of interest. David Wallon, Giovanni Castelnovo, Lisette Volpe‐Gillot, Marc Paccalin, Philippe Robert, and Vincent Camus have no conflicts of interest. Olivier Godefroy received support to his institution for attending meetings and/or travel from BRISTOL‐MYERS SQUIBB, ROCHE SAS, BIOGEN FRANCE SAS. Joël Belmin received consulting fees from Pfizer and honoraria from Novartis Pharmaceuticals. Pierre Vandel is president of the “Société Francophone de Psychogériatrie et Psychiatrie de la Personne Agée” (SF3PA) and received consulting fees from Eisai. Jean‐Luc Novella, Emmanuelle Duron, Anne‐Sophie Rigaud, Susanna Schraen‐Maschke, Bernard Sablonnière, and Audrey Gabelle have no conflicts of interest. Author disclosures are available in the supporting information.

## CONSENT STATEMENT

All participants or their legal guardians gave written informed consent.

## Supporting information

Supporting Information

Supporting Information
