# Supplementary material for: Association of caffeine consumption with cerebrospinal fluid biomarkers in mild cognitive impairment and Alzheimer's disease: A BALTAZAR cohort study
Source: Alzheimers Dement. 2024 Aug 4;20(10):6948–59. doi: 10.1002/alz.14169 (PMC11485411; doi:10.1002/alz.14169)
Supplement: Supplementary file 1 — Supporting Information [file ALZ-20-6948-s001.docx]

**Supplementary Figure 1**

**Flow-chart of the study**

**Supplementary Table 1**

**Association of CSF and plasma biomarkers at baseline with conversion to AD in MCI patients**

| **Biomarkers** | **N** | **No converters**  **N = 123** | **N** | **Converters**  **N = 24** | **Hazard ratio (95% CI)** | **P** |
| --- | --- | --- | --- | --- | --- | --- |
| CSF Tau (pg/mL) | 60 | 322 (258 to 417) | 14 | 598 (447 to 697) | 2.32 (1.54 to 3.49) | **<0.001** |
| CSF pTau181 (pg/mL) | 60 | 53 (45 to 61) | 15 | 79 (56 to 102) | 2.07 (1.42 to 3.01) | **<0.001** |
| CSF Aβ42 (pg/mL) | 54 | 866 (542 to 1221) | 13 | 579 (380 to 638) | 0.33 (0.15 to 0.72) | **0.005** |
| CSF Aβ40 (pg/mL) | 54 | 7155 (5981 to 9440) | 13 | 6795 (5854 to 8384) | 0.89 (0.49 to 1.63) | 0.72 |
| CSF Aβ42/Aβ40 (%) | 54 | 11.2 (7.6 to 15.7) | 13 | 7.0 (5.9 to 8.2) | 0.34 (0.15 to 0.71) | **0.004** |
| CSF Aβ42/pTau | 52 | 16.3 (9.0 to 25.9) | 13 | 6.5 (4.7 to 8.5) | 0.11 (0.02 to 0.44) | **0.002** |
| Pl pTau181 (pg/mL) | 110 | 2.9 ± 1.4 | 16 | 3.0 ± 1.0 | 1.10 (0.70 to 1.71) | 0.68 |
| Pl Aβ40 (pg/mL) | 110 | 272.4 ± 52.5 | 16 | 292.6 ± 64.7 | 1.51 (0.90 to 2.53) | 0.12 |
| Pl Aβ42 (pg/mL) | 110 | 40.3 ± 11.3 | 16 | 36.4 ± 10.9 | 0.71 (0.42 to 1.20) | 0.20 |
| Pl Aβ42/Aβ40 | 110 | 0.15 ± 0.04 | 16 | 0.13 ± 0.04 | 0.57 (0.33 to 0.98) | **0.041** |
| Pl NfL (pg/mL) | 67 | 16.7 (14.1 to 22.0) | 10 | 15.8 (12.2 to 22.4) | 0.84 (0.39 to 1.81) | 0.65 |

Values are presented as mean ± standard deviation or median (interquartile range, IQR). Hazard ratios are expressed for an increase of one standard deviation. Abbreviations: N: Number of available observations; CI: Confidence Interval; P: Pvalue; CSF: cerebrospinal fluid, Pl: plasma.

**Supplementary Table 2**

**Association of caffeine consumption with plasma biomarkers at baseline**

| **Plasma biomarkers** | **N** | **Low caffeine consumption**  **N = 132** | **N** | **High caffeine consumption**  **N = 131** | **Mean difference (95% CI)** | **P** |
| --- | --- | --- | --- | --- | --- | --- |
| Pl pTau181 (pg/mL) | 61 | 2.7 ± 1.1 | 62 | 3.1 ± 1.6 | -0.24 (-0.76 to 0.28) | 0.36 |
| Pl Aβ40 (pg/mL) | 112 | 271.9 ± 59.8 | 111 | 274.4 ± 58.8 | -8.6 (-24.4 to 7.2) | 0.29 |
| Pl Aβ42 (pg/mL) | 112 | 39.5 ± 12.0 | 111 | 39.1 ± 12.3 | 1.6 (-3.5 to 2.8) | 0.82 |
| Pl Aβ42/Aβ40 | 112 | 0.15 ± 0.04 | 111 | 0.14 ± 0.04 | 0.00 (-0.02 to 0.02) | 0.88 |
| Pl NfL (pg/mL) | 62 | 17.4 (13.4 ; 23.6) | 71 | 16.5 (13.9 ; 22.4) | 0.01 (-0.15 to 0.17)^1^ | 0.89^1^ |

Values are presented as mean ± standard deviation or median (interquartile range, IQR). Mean differences and pvalues were adjusted for APOEε4, age, sex, education level and tobacco consumption. ^1^ Calculated on log transformed data.
